# Oral Presentations 2

**DOI:** 10.1038/sj.bjc.6601927

**Published:** 2004-06-22


### ORAL PRESENTATIONS 2

### 2.1
### BUB1 AND AURORA COOPERATE TO MAINTAIN CHROMOSOME STABILITY

S Taylor
United Kingdom, Manchester University, Manchester

The majority of human cancer cells are aneuploid due to an underlying chromosome instability phenotype. This observation raises three questions: (1) what are the basic mechanisms which normally operate to maintain chromosome stability? (2) what aspects of these mechanisms go wrong during tumour evolution? and (3) can these defects be exploited to develop novel anti-cancer strategies? In order to address these questions, our research focuses on the spindle checkpoint.

The spindle checkpoint maintains chromosome stability by inhibiting the anaphase promoting complex (APC) until all the chromosomes correctly align on the microtubule spindle via their kinetochores. BubR1, an essential component of this checkpoint, localises to kinetochores and its kinase activity is regulated by the kinesin related motor protein Cenp-E. BubR1 also inhibits APC *in vitro,* thus providing a molecular link between kinetochore – microtubule interactions and the proteolytic machinery that regulates mitotic progression.

Several other protein kinases, including Bub1 and members of the Ipl1/Aurora family, also regulate anaphase onset. However, in human somatic cells Bub1 and Aurora kinase activity do not appear to be essential for checkpoint function: when Bub1 is repressed by RNAi or Aurora kinase activity inhibited with the small molecule ZM447439, cells arrest in mitosis when the spindle is destroyed. However, we have recently discovered that when Bub1 and Aurora are simultaneously inhibited, the checkpoint is compromised.

One explanation for this is that the checkpoint is composed of two arms, one dependent on Bub1, the other on Aurora. We suggest that both arms converge on BubR1, promoting its ability to bind APC and that these two arms respond to different spindle cues: while the Bub1 arm monitors microtubule attachment, the Aurora arm monitors biorientation. This bifurcation in the signaling mechanism may help explain why many tumor cells mount a robust checkpoint response following spindle damage despite exhibiting chromosome instability. In addition, it opens up the opportunity of developing novel "synthetic lethality" based anti-cancer strategies.

### 2.2
### SYNERGISTIC INHIBITION OF TUMOUR CELL PROLIFERATION AND UPREGULATION OF HIF-1α USING TWO ANTI-TRANSFERRIN RECEPTOR ANTIBODIES.

D.T. Jones [1], H Turley [1], P Ratcliffe [2], A.L. Harris [1]
[1] Cancer Research UK, Weatherall Institute of Molecular Medicine, Oxford, United Kingdom, [2] The Wellcome Trust Centre for Human Genetics, Oxford, United Kingdom

Iron is needed for hydroxylation of hypoxia inducible factor-1α (Hif-1α) by prolyl hydroxylases. Uptake of iron by cells is mediated by a cell surface transferrin receptor (TfR), and some tumour cells express higher levels TfR compared to normal cells. In previous studies, combinations of monoclonal antibodies (MAbs) against TfR of human tumour cells have shown antiproliferative effects. Two MAbs E2.3 and A27.15 against TfR were tested for their ability to inhibit growth of breast cancer cell line MDA-MB-468 and to induce Hif-1α and hypoxia regulated genes *in-vitro*.

Inhibition of cell proliferation was not observed after 4 days with up to 250μg/ml E2.3, while 250μg/ml A27.15 inhibited 17% cell growth. However, equal combination of E2.3 and A27.15 as low as 10μg/ml inhibited cell proliferation, with 56% inhibition of cell growth when combined MAbs were used at 250μg/ml. Synergistic upregulation of angiogenic factor VEGF was also observed when anti-TfR MAbs were combined. VEGF increased by 336% with combined 5 μg/ml MAbs after 4days, while E2.3 and A27.15 alone only increased VEGF by 19% and 35% respectively at 5μg/ml. Synergistic effect of anti-TfR MAbs was further validated by western blotting with MAbs against Hif-1α, and carbonic anhydrase IX (CAIX) a Hif-1α regulated gene. CAIX and Hif-1α were moderately upregulated by 50μg/ml of A27.15 and E2.3 when used alone, while combined anti-TfR MAbs synergistically upregulated CAIX and Hif-1α. Combined anti-TfR MAbs also downregulated the expression of TfR, while no change was observed when anti-TfR MAbs were used individually.

Treatment of breast cancer cell line with combined MAbs clearly inhibited cell growth and activated Hif-1α and Hif-1α regulated genes VEGF and CAIX synergistically. Combined anti-TfR MAbs could be used in combination with anti-Hif modulators to further inhibit cell growth *in-vitro*.

### 2.3
### ROLE OF HIF TARGET GENES BNIP3 AND CARBONIC ANHYDRASE 9 (CA9) IN HYPOXIA INDUCED CELL DEATH

AL Bacon , N Robertson , M Koukourakis , AL Harris
Cancer Research UK, Wetherall Institute of Molecular Medicine, Oxford, United Kingdom

Hypoxia, via HIF1, upregulates many genes involved in cell survival and angiogenesis. However pro-apoptotic pathways are also induced and those involved in pH regulation. We have investigated 2 such pathways that are up-regulated in most cancers, by RNA interference (RNAi) for their role in survival under hypoxia.

BNIP3 is a pro-apoptotic protein that is hypoxically up-regulated in cancer cell lines and tissues. We show that inactivation of HIF1A in MDA-435 by RNAi prevents the hypoxic up-regulation of BNIP3. While hypoxia causes reduced cell growth and increased cell death compared to normoxia, inactivation of BNIP3 reduces hypoxic induced cell death. We are exploring whether BNIP3 acts via a necrotic or apoptotic pathway and also investigating mechanisms by which cancer cells may overcome such cell death signals.

CA9 is a member of the α class of carbonic anhydrases (CAs) that catalyse the reversible hydration of $CO_2$ ($H_2O + CO_2 \Leftrightarrow H^+ + HCO_3^-$). It is over-expressed in a variety of tumour types and associated with increased metastasis and poor prognosis. RNAi was used to examine the cellular function of CA9 in MDA-468 breast carcinoma. Protein expression was significantly reduced in a dose-dependent manner, and this resulted in a 3-4 day growth delay in monolayer culture, and a 50% reduction in clonogenic survival under both normoxic and hypoxic conditions. This reduction in survival appears to be related to the role of CA9 in intracellular pH homeostasis, and makes CA9 an attractive target for cancer therapy.

Clinical application of these findings shows that in a series of 105 non small cell lung cancers treated by surgery alone, high BNIP3 was associated with poor survival and a subset had nuclear localisation of BNIP3, providing 1 mechanism for bypass of this pathway. These results suggest high levels of BNIP3 help select for an aggressive phenotype to bypass the pathway thus allowing for clonal selection.

### 2.4
### MMP-7 ACTS AS A SIGNAL FROM EPITHELIAL CELLS TO MYOFIBROBLASTS IN GASTRIC CARCINOID TUMOURS

A Varro [1], E Hemers [1], DM Pritchard [2]
[1] Dept Physiology, Univ Liverpool, Liverpool, United Kingdom, [2] Dept Medicine, Univ Liverpool, Liverpool, United Kingdom

*Background & Aims*: Prolonged elevation of the gastric hormone gastrin is virtually always associated with hyperplasia of enterochromaffin-like (ECL) cells and in some patients this progresses to gastric carcinoid tumours. The latter consist of both epithelial tissue and stroma, and both compartments may regress after removal of the gastrin-producing part of the stomach. Gastrin only acts on epithelial cells, so that these tumours provide a model for the identification of signalling pathways from epithelium to sub-epithelial cells. We previously identified increased MMP-7 expression in pre-neoplastic gastric epithelial cells; we have now examined whether MMP-7 acts as a signal from epithelial to myofibroblast cells.

*Procedures:* Gastric biopsies were obtained from patients with elevated plasma gastrin due to pernicious anaemia (PA) or gastrinoma on background of multiple endocrine neoplasia type 1 (MEN-1). MMP-7 was detected by Western blotting and immunohistochemistry; expression was studied using a promoter-reporter (luciferase) construct. Cultured myofibroblast responses to recombinant MMP-7 were studied by [$^3$H] thymidine incorporation and Western blotting of phophorylated signalling molecules.

*Results:* There was increased MMP-7 abundance in gastric biopsies of patients with PA and MEN-1, including ECL cell carcinoid tumours. In the latter, MMP-7 was localised to ECL cells but not stromal cells which were nevertheless well represented. An MMP-7 luciferase promoter reporter indicated that gastrin stimulated MMP-7 expression both directly and via COX-2. rMMP-7 stimulated the proliferation of myofibroblasts and this response was reversed by inhibitors of MEK (UO126) and PI3K (LY294002). Moreover rMMP-7 increased phosphorylation of p42/44 ERK and of Akt. *Conclusion:* MMP-7 may act as an epithelial-mesenchymal signal regulating myofibroblast function by activation of the MAPkinase and PI3kinase pathways.

© 2004 Cancer Research UK

### 2.5

### ROLE OF KERATINOCYTE GROWTH FACTOR (KGF) AND ANDROGEN IN HUMAN PROSTATIC EPITHELIAL STEM CELL DIFFERENTIATION

R Heer, AT Collins, CN Robson, HY Leung
Urology Research Group, Northern institute for cancer research, Newcastle upon Tyne, United Kingdom

**Introduction:** Understanding stem cell regulation may provide useful insight into the pathogenesis prostate cancer. It is thought that integrins play an important role in maintaining cells within stem cell compartment. We investigated the effect of blocking of the beta1 integrin function on prostate epithelial stem cells in addition to KGF and androgen, which are essential in prostate development.
Methods: Benign tissue from resected prostate samples was used to select stem cells (Collins et al., J Cell Sci 2001; 114:3865-72) which were either incubated with a blocking anti-beta1 integrin antibody, or treated with KGF (10ng/ml) or androgen analogue R1881 (10nM). Differentiation was characterised by expression of prostate acid phosphatase (PAP), androgen receptor (AR) and cytokeratin18 (CK18) using FACS analysis. Expression of alpha2 beta1 integrin was also examined.
**Results:** Blocking beta1 integrin resulted in up-regulation of PAP and CK18 (60% and 3% respecitvely, $p<0.001$) and no increase in AR levels. Treatment with KGF resulted in the down regulation of alpha2 beta1 integrin (61% decrease, $p <0.001$) and increases in PAP, CK18 and AR (35%, 36% and 39% increases respectively, $p<0.001$). R1881 treatment also resulted in the down regulation of alpha2 beta1 integrin (60% decrease, $p <0.001$) with concurrent increases in PAP, CK18, AR (33%, 36% and 34% respectively, $p<0.001$). These effects of KGF and R1881 markers of differentiation were specifically inhibited using SB 202190 (5uM) and casodex (10uM) ($p<0.05$).
**Conclusions:** Specifically blocking the beta1 integrin, induces exit from the prostate epithelial stem cell compartment as determined by the expression of PAP and CK18. KGF and androgen act to down regulate alpha2 beta1 integrin causing differentiation with the additional effect of inducing AR expression.

### 2.6

### GASTRIN INDUCED ACTIVATION OF PKB/AKT IN BARRETT'S OESOPHAGUS

JC Harris [1], PA Clarke [1], A Awan [1], J Jankowski [2], SA Watson [1]
[1] Cancer Studies Unit, University of Nottingham, Nottingham, United Kingdom, [2] Department of Cancer Biomarkers, University of Leicester, Leicester, United Kingdom

Gastrin and isoforms of the gastrin/CCK-2 receptor (including the constitutively active CCK-2Ri4sv) have previously been shown to act in an oncogenic fashion in the gastrointestinal tract, whilst activation of PKB/Akt is increasingly being seen as a key prognostic and controlling factor in carcinogenesis. This study aimed to determine a link between gastrin expression, PKB/Akt activation and subsequent IκBα degradation in the oesophageal environment, particularly in the development and progression of Barrett's oesophagus (BE).
Expression of gastrin (n=16) and CCK-2R (n=18) in normal and BE biopsy samples was quantified via real-time PCR, with immuno-staining used to confirm PKB/Akt expression (n=8). Effects of exogenous and endogenous gastrin on PKB/Akt phosphorylation and IκBα degradation in two CCK-2R positive oesophageal cell lines (OE19 and OE33) was assessed via Western blotting with *in vitro* $IC_{50}$ cytotoxicity assays used to assess cisplatin sensitivity.
*In vitro* studies demonstrated that exogenous and endogenous gastrin can stimulate PKB/Akt activation and IκBα degradation in oesophageal cell lines. CCK-2R blockade causes a reversal of this phenomenon. Gastrin or CCK-2Ri4sv mediated PKB/Akt activation was shown to confer cells with increased cisplatin resistance. Human BE biopsy samples showed a significant increase in gastrin ($p=0.0076$) and CCK-2R ($p=0.0068$) expression as well as PKB/Akt activation compared to normal tissue ($p<0.008$).
The CCK-2Ri4sv and gastrin autocrine and endocrine pathways can activate PKB/Akt as well as downstream targets *in vitro*, a mechanism potentially leading to increased resistance to cisplatin. It is suggested that the observed increase in PKB/Akt activation seen in human BE compared to normal samples could result from a similar process. We hence propose PKB/Akt as a potential prognostic marker and chemotherapeutic target in BE and oesophageal adenocarcinoma.

### 2.7

### LRP IS DIFFERENTIALLY EXPRESSED IN HUMAN GLIOBLASTOMA CELL LINES AND ITS LOW EXPRESSION IS ASSOCIATED WITH INCREASE IN ASTROCYTIC TUMOR GRADE

R Abdel-Fattah, I.M Hussaini
University of Virginia, Charlottesville, United States

Low-density lipoprotein receptor-related protein (LRP) is expressed in numerous cell-types and plays an important role in regulating proteinase activity. The receptor binds a repertoire of ligands, which have diverse biologic functions. The amount of LRP expressed on the cell surface varies between tissue types. In this study, we investigated whether LRP is differentially expressed among normal astrocytes, astrocytic tumors and different glioma cell lines (U-1242 MG, U-251 MG, U-373 MG and U-87 MG) using Western, Northern blot analyses and RT-PCR. We also determined LRP immunoreactivity in pilocytic astrocytoma, diffuse-type astrocytomas grade II/III and GBM (grade IV) using immunohistochemistry (IHC). A specific monoclonal antibody (11H4) was used against the transmembrane domain of the LRP to detect the 85-kDa band using Western blot analysis. All cell lines exhibited LRP, but expression was variable. The primary astrocytes expressed the highest level of the receptor protein followed by U-87 MG, U-1242 MG, U-251 MG and then U-373 MG cells. Expression of LRP in astrocytic cells was also confirmed by Northern blot analysis. As expected, the primary astrocytes expressed the highest level of the LRP mRNA (15 kb), suggesting that LRP expression is regulated differently in these cell lines. LRP expression using RT-PCR was in complete agreement with the Western and Northern blot analyses. Using IHC, the pilocytic astrocytoma expressed higher LRP-immunoreactivity than in astrocytoma II and III specimens or the GBM sample. We also performed Western blot analysis on frozen astrocytoma specimens and compared those with non-neoplastic astrocytes (NHA). LRP protein expression was higher in NHA and the two pilocytic astrocytomas compared to the GBM specimens. In conclusion, LRP expression decreases with increase in astrocytic tumor grade. Thus, LRP could potentially be a promising new approach for the management of malignant gliomas.

© 2004 Cancer Research UK

